# Post-COVID-19 Vaccine Parosmia: A Case Report

**DOI:** 10.7759/cureus.20292

**Published:** 2021-12-09

**Authors:** Osama S Zamzami, Abdulrahman F Kabli, Ammar S Alhothali, Omar S Alhothali, Tayil A Alharbi, Abdullah K Bahakim, Osama A Marglani

**Affiliations:** 1 Department of Medicine and Surgery, College of Medicine, Umm Al-Qura University, Al-Abdia Main Campus, Makkah, SAU; 2 Department of Otolaryngology–Head & Neck Surgery, College of Medicine, Umm Al-Qura University, Makkah, SAU

**Keywords:** astrazeneca, olfactory dysfunction, parosmia, vaccine, covid-19

## Abstract

We present the case of a healthy 38-year-old male who developed parosmia following a second dose of AstraZeneca with a negative nasal swab of coronavirus disease 2019 (COVID-19) infection. The patient noted parosmia that started suddenly after one week of receiving the second dose of AstraZeneca with no association with other symptoms. The patient has still not recovered from his parosmia until the publication of this article. The olfactory disorder was confirmed using a validated questionnaire for parosmia assessment and examination by rhinoscopy. Parosmia is a rare side effect of COVID-19, and its pathophysiological mechanism is still unknown. More research in the future is needed to know the association of parosmia with COVID-19 vaccine.

## Introduction

Coronavirus disease 2019 (COVID-19) is a global pandemic that has affected around 260,000,000 people worldwide as of November 26, 2021 [1]. This widespread morbidity and mortality precipitated rapid global vaccine development programs in history [2]. Various vaccines were developed against the virus and demonstrated effectiveness and safety [3,4]. More than 7,700,000 vaccine doses were administrated [1]. However, similar to other vaccines, they might have adverse effects caused by known and unknown mechanisms.

Olfactory dysfunction (OD) is classified into qualitative and quantitative disturbances. Quantitative OD includes anosmia and hyposmia (diminution of sensitivity). Qualitative OD is further classified into parosmia, which is defined as distorted odor perception in the presence of an odor, and phantosmia, which is described as odor perception in the absence of an apparent odor source. Both parosmia and phantosmia may occur alone but are more often associated with qualitative OD [5].

One of the most common causes of olfactory dysfunction in adults is post-viral olfactory dysfunction [6], with coronaviruses responsible for 10%-15% of all cases [7]. Several pathophysiologic mechanisms were proposed, including obstruction of the olfactory cleft, infection of the sustentacular supporting cells, injury to olfactory sensory cells via a neuropilin‐1 (NRP1) receptor, and injury to the olfactory bulb [8,9].

Despite the variety of the reported conditions related to COVID-19 vaccine, up to date, there is only one reported case in the literature on parosmia post-Pfizer-BioNTech vaccine [10]. This paper reports a unique case of parosmia post-COVID-19 vaccine (AstraZeneca) in a medically free patient.

## Case presentation

A 38-year-old male patient without any comorbid conditions consulted the rhinology clinic for a three-month history of post-COVID-19 vaccine severe parosmia. The patient reported that the parosmia started suddenly after one week of receiving the second dose of Oxford-AstraZeneca ChAd0x1 on August 31, 2021, without any other symptoms. He reported this symptom to the Ministry of Health (MOH), and they referred him to a rhinology clinic. The patient had a history of COVID-19 infection two months prior to receiving the second dose of vaccination that lasted for two weeks, and all the symptoms resolved after 10 days of COVID-19 infection. The patient had a history of smell and taste loss that lasted 7-10 days and recovered both of them completely. He did not report any smell or taste disorder over the next few months. For active infection, the patient had a negative real-time polymerase chain reaction (RT-PCR) COVID-19 test that was done right after full recovery. After vaccination, the patient reported that the foods always taste different than what they should be, and the odors that are pleasant to other people are always unpleasant for him. The patient had no phantosmia, anosmia, or hyposmia. Nasal endoscopy revealed no evidence of nasal inflammation, pus, masses, or polyps. It showed patent olfactory clefts. The clinical evaluation score for parosmia assessment was 7 out of 16 points, which indicates the presence of parosmia in our patient (Table 1) [11].

**Table 1:** Four questions addressed to facilitate the detection of the presence or absence of parosmia

Questions	This is always the case (1 point)	This is often the case (2 points)	This is rarely the case (3 points)	This is never the case (4 points)
Q1: Because of my olfactory problem, food tastes different than it should taste.	✔			
Q2: I always have a bad odor in my nose, regardless if any odor source is present.				✔
Q3: Odors that are pleasant to other people are unpleasant for me.	✔			
Q4: The biggest problem is not that I do not or weakly perceive odors, but that they smell different than they should.	✔			
Total points: 7/16	3	0	0	4

The modified Arabic Sino-Nasal Outcome Test (MA-SNOT) score of the patient was 3 out of 80 points, which indicates the absence of rhinosinusitis (Table 2) [12].

**Table 2:** Modified Arabic Sino-Nasal Outcome Test (MA-SNOT) score

Items	No problem (0)	Very mild problem (1)	Mild or slight problem (2)	Moderate problem (3)	Severe problem (4)	Problem as bad as it can be (5)	Most important symptoms
1. Need to blow nose	✔						
2. Sneezing	✔						
3. Runny nose	✔						
4. Blockage/congestion of the nose	✔						
5. Loss of sense of taste/smell			✔				
6. Cough	✔						
7. Itching of the nose, throat, or eyes	✔						
8. Postnasal discharge (dripping at the back of the nose)	✔						
9. Ear fullness	✔						
10. Facial pain/pressure	✔						
11. Lack of a good night’s sleep	✔						
12. Waking up tired	✔						
13. Fatigue		✔					
14. Double vision	✔						
15. Weak vision	✔						
16. Exophthalmos	✔						
Total: 3/80	0	1	2	0	0	0	

The patient received five-day oral dexamethasone 4 mg, nasal spray fluticasone twice daily, daily oral omega-3, seven-day oral omeprazole 20 mg, olfactory rehabilitation, and follow-up. Consent was obtained orally and signed by the patient himself.

## Discussion

COVID-19 infection has become one of the causes of parosmia recently. Many studies discuss the long-term complication of COVID-19. The study of Ohla et al. included 1468 out of 12,313 patients who reported a loss of smell and taste after the infection and full recovery of their sense of smell [13]. In the UK, as listed in the COVID‐19 Oxford University/AstraZeneca Vaccine Analysis Print, 402 out of 842,270 cases are suspected of having parosmia after receiving the vaccine [14]. Until now, no case report has been published on post-AstraZeneca vaccine parosmia. A similar case was reported, in which parosmia presented after the second injection of the Pfizer vaccine. In the study of Lechien et al., they reported six patients with different scenarios [10]. They presented with alternation of smell after receiving vaccines. Two of them reported hyposmia, while two others reported anosmia. One patient reported parosmia, and the last one complained of dysgeusia without smell disorder [10]. The patient who complained of parosmia is a 33-year-old female who presented with the condition after 9-14 days after receiving the second dose of the Pfizer vaccine, with other symptoms such as arthralgia and fatigue with a negative nasal swab. Our patient is a 38-year-old male with a three-month history of parosmia after receiving the second dose of AstraZeneca. No anosmia or other symptoms were reported. He was a healthy male subject and nonsmoker and had no history of nasal disease or previous nasal surgery. The patient had a history of loss of smell during COVID-19 infection, which lasted for 7-10 days, and recovered the smell completely. In evaluating the patient's clinical picture, we used questionnaire-based scores to assess his parosmia objectively, as shown in Table 1 [11]. The total score of the four questions was 7 out of 16 points, which indicates the presence of parosmia. Also, we used a validated questionnaire to assess if the patient has symptoms of rhinosinusitis [12]. The modified Arabic Sino-Nasal Outcome Test (MA-SNOT) score was 3 out of 80 points, as shown in Table 2 [12]. The patient has only mild loss of sense of taste/smell and very mild fatigue. This means that the patient does not have rhinosinusitis as a potential cause of his parosmia. In addition, more research has to be done to determine the prevalence and association of parosmia after COVID-19 vaccine, regardless of the type of vaccine.

## Conclusions

Olfactory and taste dysfunction are common symptoms of COVID-19 infection, but their association to COVID-19 vaccine is rare. The pathophysiological mechanism is not yet understood. Still, COVID-19 vaccines have benefits that outweigh this rare side effect. We aimed to report this case to increase physician awareness of the possibility of post-COVID-19 vaccination parosmia. Future research is needed to further investigate parosmia association with COVID-19 vaccine.
